# Supplementary material for: Pre-lacteal feeding practices and associated factors among mothers of children aged less than 12 months in Jinka Town, South Ethiopia, 2018/19
Source: PLoS One. 2020 Oct 13;15(10):e0240583. doi: 10.1371/journal.pone.0240583 (PMC7553318; doi:10.1371/journal.pone.0240583)
Supplement: S1 Questionnaire — (DOCX) [file pone.0240583.s002.docx]

| **Part 1**: **Socio-demographic variables of Mother and child** | | | | |
| --- | --- | --- | --- | --- |
| S. no | Question | answer | code | Skip |
| 101 | How old are you? | ---------------------- |  |  |
| 102 | Family size | ------------------ |  |  |
| 103 | What is your current marital status? | 1.Single 2.Married 3.Divorced 4.Widowed |  |  |
| 104 | What is your level of education? | 1. Unable to read and write  2.Primary education 3.Secondary education and above |  |  |
| 105 | What is your religion? | 1. Orthodox 2. Protestant 3. Muslim 4. Other (specify)----- |  |  |
| 106 | Which ethnic group do you belong to? | 1. Amhara 2. Ari 3. Gofa 4. Wolayta  5. Basketo 6. Others (specify)---- |  |  |
| 107 | What is your current occupation? | 1. Student 2. Private employee  3. Civil servant 4. Daily laborer  5. Trader 6. Farmer  7. House wife 8. Other (Specify))----- |  |  |
| 108 | What is the educational status of your husband? | 1. Unable to read and write  2.Primary education 3.Secondary education and above |  |  |
| 110 | Gender of your (the index) child? | 1. Male 2. Female |  |  |
| 111 | Birth order of the index child | ----------------- |  |  |
| 112 | Birth spacing with the previous child | ------------------ |  |  |
| 113 | Number of children | -- |  |  |
| 114 | What is the approximate household income from all the sources per month? | ------------------- in Eth birr |  |  |
| 115 | Privet home | 1 yes  2 no |  |  |
| 116 | A radio | 1 Yes  2 no |  |  |
| 117 | A television | 1 yes  2 no |  |  |
| 118 | A mobile phone | 1 yes  2 no |  |  |
| 119 | A table | 1 yes  2no |  |  |
| 120 | A chair | 1 yes  2 no |  |  |
| 121 | A bed with cotton/ sponge/spring matters | 1 yes  2 no |  |  |
| 122 | A kerosene lamp/pressure lamp | 1 yes  2 no |  |  |
| 123 | Does household own any agricultural | 1 yes  2 no |  |  |
| 124 | How many (local units) of agricultural land do you own? | -----------(local unit) |  |  |
| 125 | Annual farm product per quintal | ----------------Quintal-. |  |  |
| 126 | Wheat | ----------------Quintal-. |  |  |
| 127 | Maze | ----------------Quintal-. |  |  |
| 128 | Barley | ------------------Quintal. |  |  |
| 129 | Presence of cattle’s domestic animals | 1. Yes  2. No |  |  |
| 130 | Milk cows, oxen or bulls | ------------- in no |  |  |
| 131 | Horses, donkeys, or mules | ------------- in no |  |  |
| 132 | Goats? | -------------- in no |  |  |
| 133 | Sheep? | -------------- in no |  |  |
| 134 | Chickens? | -------------- in no |  |  |
| 135 | Beehives? | -------------- in no |  |  |
| **Part 2: Infant feeding practices** | | | | |
| S no | question | Answer | code | Skip |
| 201 | Did you give anything to drink and/or eat before breast milk within 3 days for your child, after delivery? | 1.Yes 2.No |  | If ,no skip to ques 205 |
| 202 | If question 16 is yes, what did you give? (Multiple responses is possible) | 1. Plain water 2.Glucose water 3. Cow milk 4. Water  5. Butter 6. Formula milk 7. Other (specify)----- |  |  |
| 203 | Why did you give anything to drink and/or eat before breast milk after delivery? (Multiple responses Is possible) | 1. Breastfed for newborns will be thirsty 2. Breast problem 3. Maternal medical illness 4. Inadequate milk secretion 5. Infant feeding problem 6. For child growth 7. Cultural practice 8. To clean infant’s bowel/throat/mouth 9. Other (specify) |  |  |
| 204 | Who influenced you to give PLF for your child? | 1. My own decision 2. Grandparents 3. TBA 4. Husband 5. Friends 6. Others specify------- |  |  |
| 205 | Did you feed colostrum (the first yellow milk) for this index during the first five days after birth? | 1.yes 2.no |  |  |
| 206 | When did you initiate breast feeding (name of the index child) after birth? | ----------------- |  |  |
| 207 | If question 205 is no, why you avoid colostrum for your child? (Multiple responses is possible) | 1. Maternal medical illness 2. For the child growth 3. My breast has no milk 4. Cause Abdominal discomfort and diarrhea 5. Other (specify)------- |  |  |
|  | **Part 3: Maternal health services related variables** | | | |

| S no | questions | | answers | code | | | Skip |  |
| --- | --- | --- | --- | --- | --- | --- | --- | --- |
| 301 | Did you attend the ANC clinic during your last pregnancy? | | 1. Yes 2. No |  | | | If no jump to quest no 303 |  |
| 302 | If Yes, how many times did you attend ANC clinic during the last pregnancy? | | ------------------ |  | | |  |  |
| 303 | Did you get breastfeeding counseling for the index child? | | 1. Yes 2. No |  | | | If no jump to quest no 305 |  |
| 304 | If yes, what were you told about breast feeding? | | 1. Benefits of breastfeeding 2. Positioning of the baby 3. Exclusive breastfeeding 4.Management of breast problem 5. Expression of breast milk |  | | |  |  |
| 305 | How many times do you give live birth? | | ------------------- |  | | |  |  |
| 306 | Where did you give birth? | | 1. Health facility 2. At home 4. TBA’s place 5. Other (specify) ------------- |  | | |  |  |
| 307 | What was the mode of delivery? | | 1. CS delivery 2. Normal spontaneous delivery 3. Instrumental delivery 4. Other specify------------- |  |  |  |  |  |
| 308 | Who delivered you? | | 1. Health professionals 2. Traditional birth attendant 3. Others specify------------- |  | |  | | |
| 309 | Did you receive PNC following delivery of your index child | | 1 yes 2 no |  | |  | | |
| **Part 4: Misconception assessment questions** | | | | | | | | |
| 309 | Do you belief pre lacteal feeding is important for your child? | 1. YES 2. No | | If no jump to quest no 311 |  |  |  |  |
| 310 | If yes, could you mention? | 1. For child health and growth 2. Breastfed for newborns will be thirsty 3. To calm/soothe the baby 4.To clean infants bowel/throat/mouth  5.If another (specify)-------- | |  |  |  |  |  |
| 311 | Do you have previous experience of PLF | 1 Yes 2. No | |  |  |  |  |  |
| 311 | Do you know the risks associated with PLF? | 1.Yes 2.No | |  |  |  |  |  |
| 312 | If yes, could you mention? (Multiple responses is possible) | 1.Diarrhea  2. Poor growth 3. Infection 4. Vomiting  5.If another (specify)-------- | |  |  |  |  |  |
|  | | | | |  |  |  |  |

| **Part 5: Breast feeding knowledge assessment question** | | | | |
| --- | --- | --- | --- | --- |
| 401 | Breastfeeding is important for infant health? | 1 true 2 false | Code | Skip |
|  | Breastfeeding is important for maternal health? | 1 true 2 false |  |  |
|  | An infant should be put to breast immediately after birth? | 1 true 2 false |  |  |
|  | The first milk/colostrum should be given to an infant? | 1 true 2 false |  |  |
|  | Prelacteal feeding is not needed for an infant before starting breast milk? | 1 true 2 false |  |  |
|  | Breast milk alone without water and other liquids is enough for an infant during the first 6 months of life? | 1 true 2 false |  |  |
|  | Starting from 6 month an infant should start complementary feeding and continued breastfeeding up to 2 years and beyond? | 1 true 2 false |  |  |

**Part 6: Husbands support for breastfeeding practice**

| 501 | Encourage on health service utilization | 1 yes 2 no | Code | Skip |
| --- | --- | --- | --- | --- |
|  | encourage immediate initiation of breast feeding after birth | 1 yes 2 no |  |  |
|  | encourage colostrum feeding | 1 yes 2 no |  |  |
|  | discourage prelacteal feeding | 1 yes 2 no |  |  |
|  | encourage BF on demand day and night | 1 yes 2 no |  |  |
|  | encourage EBF for six months | 1 yes 2 no |  |  |
|  | psychologically supporting | 1 yes 2 no |  |  |
|  | do direct child care | 1 yes 2 no |  |  |
|  | do other house hold chores | 1 yes 2 no |  |  |
|  | provide additional food for BF mother | 1 yes 2 no |  |  |
|  | encourage to BF in front of others/in public places | 1 yes 2 no |  |  |

**Amharic Questionnaires (የአማርኛ መጠይቅ)**

**ክፍል 1 ፡*-* እናቶች እና ህፃናት ማህበራዊና አኗኗር ሁኔታዎች መረጃ**

| ተ.ቁ | ጥያቄዎች | ምላሾች | | ኮድ | ዝለል |
| --- | --- | --- | --- | --- | --- |
| 101 | እድሜሽ ስንት ነው? | 1. _________ ዓመት 2. አላውቀውም | |  |  |
| 102 | ቤተሰብ ብዛት? | -------------- | |  |  |
| 103 | የጋብቻ ሁኔታ? | 1. ያገባች 2. ያላገባች 3. የተፋታች 4. የሞተባት | |  |  |
| 104 | የት/ት ሁኔታ? | 1. ማንበብና መፃፍ የማትችል 2. የ1ኛ ደረጃ ት/ት የተማረች 3. የ2ኛ ደረጃ ት/ት ና ከሳ በላይ | |  |  |
| 105 | ሃይማኖት? | 1. ኦርቶዶክስ ተዋህዶ 2. ፕሮቴስታንት 3. ሙስሊም 4. ሌላ (ጥቀሺ)--------- | |  |  |
| 106 | ብሔር? | 1. አማራ 2.አር 3.ጎፋ  4. ዎላይታ 5.ባስኬቶ 5. ሌላ (ጥቀሺ)--------- | |  |  |
| 107 | የስራ ሁኔታ? | 1. ተማሪ 2. የግል ተቀጣሪ 3. የመንግስት ተቀጣሪ 4. የቀን ሠራተኛ 5. ነጋዴ 6. ገበሬ  7. የቤት እመቤት 8. ሌላ (ጥቀሺ)------ | |  |  |
| 108 | የባልሽ ት/ት ሁኔታ? | 1. ማንበብና መፃፍ የማችል  2. የ1ኛ ደረጃ ት/ት የተማረች 3. የ2ኛ ደረጃ ት/ት ና ከሳ በላይ | |  |  |
| 110 | የህፃኑ ፆታ? | 1. ወንድ 2. ሴት | |  |  |
| 111 | ህፃኑ ስንተኛ ልጅ ነው? | --------ኛ | |  |  |
| 112 | ከዚህ በፊት ከተወለደው ህፃን ጋር ያለው የእድሜ መራራቅ? | ------------- | |  |  |
| 113 | የልጆች ብዛት? | ----------- | |  |  |
| 114 | ወርሃዊ ገቢሽ ምን ያህል ነው? | _________የኢትዮ. ብር | |  |  |
| 115 | የግል መኖርያ በት አላችሁ? | 1. አዎ 2. አይደለም | |  |  |
| 116 | ሬድዮ አላችሁ? | 1. አዎ 2. አይደለም | |  |  |
| 117 | ቴሌቪዥን አላችሁ? | 1. አዎ 2. አይደለም | |  |  |
| 118 | ተንቀሳቃሽ ስልክ አላችሁ? | 1. አዎ 2. አይደለም | |  |  |
| 119 | ጠረጴዛ አላችሁ? | 1. አዎ 2. አይደለም | |  |  |
| 120 | ወንበር አላችሁ? | 1. አዎ 2. አይደለም | |  |  |
| 121 | ጥጥ(ስፓንጅ)ስፕሪንግ ፍራሽ ያለው አልጋ አላችሁ? | 1. አዎ 2. አይደለም | |  |  |
| 122 | በጋዝ የሚሰራአምፖል(ኩራዝ)አላችሁ? | 1. አዎ 2. አይደለም | |  |  |
| 123 | ከቤተሰቡ አባላት ውስጥ  የእርሻ መሬት ያለው ይኖራል? | 1. አዎ 2. አይደለም | |  |  |
| 124 | አለ ካሉ የእርሻ መሬቱ ስፋት ምን ያህል ይሆናል?መጠኑን ይግለጹ? | ----------- | |  |  |
| 125 | በአመት በእርሻ ምን ያህል ምርት ያገናሉ | ----------- ኩንታል | |  |  |
| 126 | በስንደ | ----------- ኩንታል | |  |  |
| 127 | በበቆሎ | ----------- ኩንታል | |  |  |
| 128 | በገብስ | ----------- ኩንታል | |  |  |
| 129 | በቤት ውስጥ የሚኖሩ ለእርሻ ወይም ለእርባታ የሚውሉ የቤት እንስሳት ይኖራሉ? | 1. አዎ 2. አይደለም | |  |  |
| 130 | የወተትላሞች፣በሬዎች፣ ጥጃ | 1. አዎ 2. አይደለም በቁጥር---------- | |  |  |
| 131 | ፈረሶች፣ አህያዎች፣ በቅሎ | 1. አዎ 2. አይደለም በቁጥር---------- | |  |  |
| 132 | ፍየሎች | 1. አዎ 2. አይደለም በቁጥር---------- | |  |  |
| 133 | በጎች | 1. አዎ 2. አይደለም በቁጥር---------- | |  |  |
| 134 | ዶሮዎች | 1. አዎ 2. አይደለም በቁጥር---------- | |  |  |
| 135 | የንብ ቀፎ | 1. አዎ 2. አይደለም በቁጥር---------- | |  |  |
|  | | |  |  |  |

**ክፍል *2*፡*-* የህፃኑ የአመጋገብ ሁኔታ**

| ተ **.ቁ** | ጥያቄዎች | ምላሾች | ኮድ | ዝለል | | |
| --- | --- | --- | --- | --- | --- | --- |
| 201 | ህፃኑ ወዲያው እንደተወለደ ከጡት ወተት በፊት የሚበላ ወይም የሚጠጣ ነገር ሠጥተሸው ነበር? | 1. አዎ 2. አይደለም |  | 201 አይደለም ከሆኔ ዋደ 205 ዝለል | | |
| 202 | ለጥያቄ ቁጥር 201 መልስሽ አዎ ከሆነ ምን ሠጠሸው | 1. ውሃ 2. በውሃ የተበጠበጠ ስኳር 3. የላም ወተት 4. ቅቤ 5. የዱቄት ወተት 6. ሌላ ካለ (ጥቀሽ)-------- |  |  | | |
| 203 | ለምን ለህፃኑ ወዲያው እንደተወለደ ከጡት ወተት በፊት የሚበላ ወይም የሚጠጣ ነገር ሠጠሸው? (ከአንድ በላይ ምላሽ ይቻላል) | 1. የጡት ወተት መስጠት ህፃኑን ስለሚጠማው 2. . ጡት የማጥባት ችግር ስለገጠመኝ 3. እናት ህመም ስለገጠማት  4. ጡቴ በቂ ወተት ስላልነበረው 5 ህፃኑ ስለታመመ 6. ለህፃኑ እድገት 7. በአካቢበው ባህል ስለሆነ 8. የህፃኑን አፍ፣ ጉሮሮና አንጀት እንዲያጠራው 9. ሌላ ካለ (ጥቀሽ)---- |  |  | | |
| 204 | ለህፃኑ ከጡት በፊት የሚበላ ወይም የሚጠጣ ነገር እንድትሠጪው ማን መከረሽ? | 1. የራሴ ውሳኔ ነበር 2. አያቴ 3. የልምድ አዋላጅ 4. ባለቤቴ 5. ጓደኞቼ 6ሌላ ካለ (ጥቀሽ)------ |  |  | | |
| 205 | ህፃኑ በተወለደ በመጀመሪያዎቹ አምስት ቀናት እንገር (የመጀመሪያው እና ቢጫው የወተት ጡት) አጥብተሸው ነበር? | 1. አዎ 2. አይደለም |  |  | | |
| 206 | ከወለድሽ በኋላ ህፃኑን የጡት ወተት ማጥባት መች ጀመርሽ? | ……………………. |  |  |  |  |
| 207 | ለጥያቄ ቁጥር 20 መልስሽ አይደለም ከሆነ ለምን ለህፃኑ እንገር (የመጀመሪያውና ቢጫው የጡት ወተት) መስጠት ከለከልሽ? (ከአንድ በላይ ምላሽ ይቻላል) | 1. እናት ህመም ስለገጠማት 2. ለህፃኑ እድገት ስለሚበጅ 3. ጡቴ በቂ ወተት ስላልነበረው 4. የሆድ ህመም እና ተቅማጥ ስለሚያስከትል  5. ሌላ ካለ (ጥቀሽ)--------- |  | |  |  |
|  | | |  |  |  |  |

**ክፍል *3*፡የእናቶች ጤና አገልግሎት በተመለከተ**

| ተ. ቁ | ጥያቄዎች | ምላሾች | | | ኮድ | ዝለል | |  |
| --- | --- | --- | --- | --- | --- | --- | --- | --- |
| 301 | በእርግዝናሽ ጊዜ የቅድመ ወሊድ ክትትል በጤና ማዕከል ተከታትለሽ ነበር? | 1. አዎ 2. አይደለም | | |  | 301 አይደለም ከሆኔ ዋደ 303 ዝለል | |  |
| 302 | ለጥያቄ ቁጥር 301 መልስሽ አዎ ከሆነ ምን ያህል ጊዜ የቅድመ ወሊድ ክትትል አደረግሽ? | ---------------- | | |  |  | |  |
| 303 | ስለ ጡት ማጥባት ምክር/ መረጃ አግኝተሸ ያውቃሉ ? | 1. አዎ 2. አይደለም | | |  | 303 አይደለም ከሆኔ ዋደ 305 ዝለል | |  |
| 304 | ለጥያቄ ቁጥር 303 መልስሽ አዎ ከሆነ ስለ ጡት ማጥባት ምን ተነገረሽ? | 1. ስለ ጡት ማጥባት ጥቅም 2. የህፃኑ አቀማመጥ በጡት ማጥባት ጊዜ 3. ለ6 ወር የእናት ጡት ብቻ ስለመስጠት 4. ስለ ጡት ማጥባት ችግርና መፍትሄው 5. የጡት ወተት አልቦ ስለመስጠት 6. ሌላ ካለ (ጥቀሺ)------- | | |  |  | |  |
| 305 | በህይወት ያለ ህፃን ምን ያህል ጊዜ ወለድሽ? | -------------------- | | |  |  | |  |
| 306 | ልጅዎን የት ወለዱት? | 1. በመንግስት የጤና ማዕከል 2. በግል ክሊኒክ 3. በቤት ውስጥ 4. የልምድ አዋላጆች ያሉበት  5. ሌላቦታ ካለ (ጥቀሺ) | | |  |  | |  |
| 307 | ልጅዎን የወለዱት በምን መልኩ ነው? | 1. በሆዴ በኩል የቀዶ ጥገና ተደርጎልኝ 2. በማህፀን በኩል ያለምንም መሣሪያ 3. ሌላ ካለ (ጥቀሺ)--------- | | |  | |  |  |
| 308 | ልጅዎን ማን አዋለድዎት? | 1. የጤና ባለሙያዎች 2. የልምድ አዋላጆች  3. ሌላ ካለ (ጥቀሺ)--------- | |  | | |  | |
| 309 | ድህረወሊድ ክትትል ተከታትለሽ ነበር? | 1. አዎ 2. አይደለም | |  | | |  | |
| **ክፍል *4*፡የእናቶች ከጡት ወተት በፊት የሚበላወይም የሚጠጣ ነገር ላይ ያላቸዉ እምነት በተመለከተ** | |  | | | | | | |
|  | |  |  |  |  |  |  |  |
| 401 | ከጡት ወተት በፊት የሚበላ ወይም የሚጠጣ ነገር ለህፃኑ ጠቃሚ ነዉ ብለዉ ያምናሉ? | 1. አዎ 2. አይደለም |  | | | | 309 አይደለም ኬሆኔ ዋደ 311 ዝለል | |
| 402 | ለጥያቄ ቁጥር 309 መልሱን አዎ ካሉ ሊጠቅሱይችላሉ? (ከአንድ በላይ ምላሽ ይቻላል) | 1. ለህፃኑ ጤንነት 2. ለህፃኑ እድገት 3. ህፃኑ እንዳይጮህ /እንዳያለቅስ ለማድረግ/ 4. የህፃኑን አፍ፣ ጉሮሮና አንጀት እንዲያጠራው ለማድረግ  5. ሌላ ካለ (ጥቀሺ)----- |  | | | |  | |
| 403 | ከዚህ በፊት ለተወለደው ህፃኖች ከጡት ወተት በፊት የሚበላ ወይም የሚጠጣ ነገር ሠጥተሸው ነበር? | 1. አዎ 2. አይደለም |  | | | |  | |
| 404 | ከጡት ወተት በፊት የሚበላ ወይም የሚጠጣ ነገር ሊያስከትለው ስለሚችል ነገር ያውቃሉ? | 1. አዎ 2. የለም |  | | | |  | |
| 405 | ለጥያቄ ቁጥር 311 አዎ ካሉ ሊጠቅሱ ይችላሉ? | 1. ተቅማጥ ያስከትላል 2. የህፃናኑን እድገት ያቀጭጫል 3. ኢንፌክሽን ያስከትልላል 4. ትውከት ያስከትላል 5. ሌላ ካለ (ጥቀሺ) |  | | | |  | |

**ክፍል *5*፡የእናቶች የጡት ማጥባት እዉቀት በተመለከተ**

| 501 | ጡት ማጥባት ለህፃኑ ጤንነት አስፈላጊ ነዉ፡፡ | 1.ትክክል 2. ሀሰት | ኮድ | ዝለል |
| --- | --- | --- | --- | --- |
|  | ጡት ማጥባት ለእናት ጤንነት አስፈላጊ ነዉ፡፡ | 1.ትክክል 2. ሀሰት |  |  |
|  | ህፃኑ ወዲያው እንደተወለደ የጡት ወተት መጥባት አለበት፡፡ | 1.ትክክል 2. ሀሰት |  |  |
|  | ህፃኑ በተወለደ በመጀመሪያዎቹ አምስት ቀናት እንገር (የመጀመሪያው እና ቢጫው የወተት ጡት)መጥባት አለበት፡፡ | 1.ትክክል 2. ሀሰት |  |  |
|  | ህፃኑ ወዲያው እንደተወለደ ከጡት ወተት በፊት የሚበላ ወይም የሚጠጣ ነገር አያስፈልግም፡፡ | 1.ትክክል 2. ሀሰት |  |  |
|  | የጡት ወተት ብቻ ለህፃኑ እስከ 6 ዎር በቂ ነዉ፡፡ | 1.ትክክል 2. ሀሰት |  |  |
|  | ከ6 ዎር ጀምሮ ለህፃኑ ተጨማር ምግብ መጀመርና የጡት ወተት እስከ ሁለት አመት ማጥባት አስፈላጊ ነዉ፡፡ | 1.ትክክል 2. ሀሰት |  |  |

**ክፍል *6*፡የባል ድጋፍ በተመለከተ**

| 601 | የጤና አገልግሎት እንድትጠቀም ያበረታታል | 1.አዎ 2. አይደለም | ኮድ | ዝለል |
| --- | --- | --- | --- | --- |
|  | ህፃኑ ወዲያው እንደተወለደ የጡት ወተት እንድትሰጪ ያበረታታል | 1.አዎ 2. አይደለም |  |  |
|  | ህፃኑ በተወለደ የመጀመሪያው እና ቢጫው የጡት ወተት እንድትሰጪ ያበረታታል | 1.አዎ 2. አይደለም |  |  |
|  | ህፃኑ ወዲያው እንደተወለደ ከጡት ወተት በፊት የሚበላ ወይም የሚጠጣ ነገር እንዳትሰጪ ያደናቅፋል | 1.አዎ 2. አይደለም |  |  |
|  | ህፃኑ የጡት ወተት የምፈልግበት ግዘ ቀንና ማታ እንድትሰጪ ያበረታታል | 1.አዎ 2. አይደለም |  |  |
|  | የጡት ወተት ብቻ ለህፃኑ እስከ 6 ዎር እንድትሰጪ ያበረታታል | 1.አዎ 2. አይደለም |  |  |
|  | የስነልቦና ድጋፍ ያደርጋል | 1.አዎ 2. አይደለም |  |  |
|  | ህፃኑን ያንከባክባል | 1.አዎ 2. አይደለም |  |  |
|  | ሌላ የቤት ስራዎችን ያግዛል | 1.አዎ 2. አይደለም |  |  |
|  | ለህፃኑ የጡት ወተት ስትሰጪ ተጨማሪ ምግብ ያቀርባል | 1.አዎ 2. አይደለም |  |  |
|  | ሰዎች ባሉበት ለህፃኑ የጡት ወተትእንድትሰጪ ያበረታታል | 1.አዎ 2. አይደለም |  |  |
