# Supplementary material for: Pre-lacteal feeding practices and associated factors among mothers of children aged less than 12 months in Jinka Town, South Ethiopia, 2018/19
Source: PLoS One. 2020 Oct 13;15(10):e0240583. doi: 10.1371/journal.pone.0240583 (PMC7553318; doi:10.1371/journal.pone.0240583)
Supplement: Attachment — Submitted filename: Response for editor and reviewers for Prelacteal feeding.docx [file pone.0240583.s003.docx]

Dear Editor and Reviewer

Sincerest thanks for your response and reviewers comments on our manuscript. We have modified the paper in response to the extensive and insightful editor and reviewer comments. We would be glad to respond to any further questions and comments that you may have.

Responses

For Reviewer 1

- It is appropriate to order the percentage of the variables from bigger to smaller in the frequency table?

Of course many literatures don’t give attention but to get the reader’s attention we understand to present systematically and we have modified according to the comment

- You can emphasize why this study was conducted in this specific region such as ethnicity, low educational level of the mothers, etc. Also, please this explanation in introduction and discussion sessions. We amend it accordingly
- The other comments are modified accordingly

For Reviewer 2

- Key words…. Modified accordingly
- Line 24 modified according to the comment
- Line 39 modified according to the comment
- Line 47 WHO, Modified by World Health Organisation (WHO).
- Line 47-49 WHO recommends Modified according to the comment
- Line 161-173 editing, Edited
- Line 170-71,
- Multi co linearity test was carried out to see the correlation between all independent variables using collinearity statistics which is tolerance > 0.1 and variance inflation factor < 10. If two of the variables are highly correlated, then this may be the possible source of multicollinearity. Of course standard error is also used for checking multicollinearity, in our case standard error is also less than 4, and moreover, multicollinearity is a state of very high inter-correlations or inter-associations among the independent variables. It is therefore multicollinearity can also be detected with the help of tolerance and its reciprocal, called variance inflation factor (VIF).
- Line 213-15 were these problems self-diagnosed or diagnosed by health professionals? Diagnosed by health professionals
- Line 218: “purported” does not seem to be the best word. Need to explain what is meant by it’s use.

It was to say there belief on the aim/advantage of pre-lacteal feeding, just we removed the word since we also understand it is not the appropriate term

- Table 3: was “don’t know” a response option? Or were these “true/false” responses?

It was True/false response and modified in the table also since the questionnaire was prepared as true/false option

- Table 4: see comment on collinearity. Indicate p-values for unadjusted ORs

Thank you the comment, Pvalue was provided in the foot note

- Lines 277-86: explain why the age of the child might make a difference in prelacteal feeding – recall? communication campaign? Similarly for other comparisons – clarify possible reasons for differences.

Mothers having children <24 months may face difficulties to remember what they fed their child. Also, it is assumed that mothers with good educational status have a high chance for visiting health centers. It is modified accordingly

- Please provide rates of breastmilk substitute feeding. Breastmilk substitutes are important prelacteal feeds that don’t seem to have been considered in the study. This may explain the contradiction with the Egypt study (lines 294-97)

The pre-lacteal feeds found in this study were Plain water (22 mothers), glucose water (3 mothers), cow milk (3 mothers), butter (13 mothers), Tenadam (11 mothers)

- Are more educated mothers giving breast milk substitutes?

In our study mothers with good educational status were feeding their child breast milk as the odds of providing pre-lacteal feeding among mothers who were unable to read and write were nearly five folds higher than those who were secondary and above educational level, where as in Egypt study they even recommend that Further education of the mothers and health staff about adverse effects of PLF is required as they thought those well-educated are poor in practicing exclusive breast feeding based on their data.

- The discussion is quite superficial. It should explore explanations in greater depth. For example, lines 306-13 – is anything known about the content of breastfeeding counselling in the different studies? The contents are similar throughout the country but there is variation in practicing the counseling during ANC , delivery and Post natal period
- Describe “traditional beliefs that favour prelacteal feeding” (lines 319-20).
   Described according to the comment
  For Reviewer 3
- Even though prevalence is around not much more than 10%, and the results would likely be similar, it would be interesting to see prevalence ratios instead of odds ratios.

The prevalence of pre-lacteal feeding practice in this study was 53 (12.6%)

- Sample size calculation: it is not clear why the design effect was considered. Was the clustering of children by area (Kebeles)? Yes there was clustering in the kebele (urban kebele and rural kebele) and we used design effect of 1.5 to consider the loss of effectiveness by the use of cluster sampling, instead of simple random sampling.
- Also, it seems like since the goal was to estimate prevalence, the authors should have calculated a required SS to estimate the prevalence with a particular precision. What does “95% certainty and maximum discrepancy of 5% between122 the sample size and the underlining population” mean?

This was to mean the sample size was calculated by assuming 95% confidence level and a margin of error of 5%

- What are the 3 breastfeeding knowledge questions? These should be listed in a table or appendix even if a reference is given.

It is provided in the supplementary information with the tools

- Multicollinearity – there needs to be clarification of how this was done. Standard error or R-square? Multi-collinearity test was carried out to see the correlation between all independent variables using collinearity statistics which is tolerance > 0.1, variance inflation factor < 10 and standard error which was less than 4
- In the decisions for pre-lacteal feeding section, what are the denominators of the listed percentages? Looks like they are for the N=53 who practiced pre-lacteal feeding. Throughout, the n (numerator and denominator) has to be clearly stated

Thank you for the comment and yes it is from N=53 and modified accordingly
