# Supplementary material for: Pre-lacteal feeding practices and associated factors among mothers of children aged less than 12 months in Jinka Town, South Ethiopia, 2018/19
Source: PLoS One. 2020 Oct 13;15(10):e0240583. doi: 10.1371/journal.pone.0240583 (PMC7553318; doi:10.1371/journal.pone.0240583)
Supplement: Attachment — Submitted filename: Dear editor and reviewers.docx [file pone.0240583.s004.docx]

Dear Editor and Reviewer

Sincerest thanks for your response and reviewers comments on our manuscript. We have modified the English language of this paper in response to the comments given. We would be glad to respond to any further questions and comments that you may have.
